# Efficacy and safety of brolucizumab versus aflibercept in eyes with early persistent retinal fluid: 96-week outcomes from the HAWK and HARRIER studies

**DOI:** 10.1038/s41433-022-02092-5

**Published:** 2022-05-21

**Authors:** David R. Lally, Anat Loewenstein, Jennifer J. Arnold, Yit C. Yang, Kinfemichael Gedif, Catherine Best, Hersh Patel, Ramin Tadayoni, Jeffrey S. Heier

**Affiliations:** 1 New England Retina Consultants, Springfield, MA USA; 2 Department of Surgery, University of Massachusetts Medical School-Baystate, Springfield, MA USA; 3 Tel Aviv Sourasky Medical Center, Sackler Faculty of Medicine, Tel Aviv University, Tel Aviv, Israel; 4 Marsden Eye Specialists, Parramatta, NSW Australia; 5 Royal Wolverhampton Hospitals NHS Trust, Wolverhampton, UK; 6 Novartis Pharmaceuticals Corporation, Fort Worth, TX USA; 7 Novartis Pharma AG, Basel, Switzerland; 8 Novartis Pharmaceuticals Corporation, East Hannover, NJ USA; 9 Department of Ophthalmology, Université de Paris, AP-HP, Lariboisière, Saint Louis and Rothschild Foundation Hospitals, Paris, France; 10 Ophthalmic Consultants of Boston, Boston, MA USA; 11 Present Address: IVERIC bio, Inc., New York, NY USA

**Keywords:** Macular degeneration, Prognostic markers

## Abstract

**Objective:**

Post-hoc analysis to compare the outcomes of brolucizumab 6 mg vs. aflibercept 2 mg in neovascular age-related macular degeneration (nAMD) patients with early persistent retinal fluid in HAWK and HARRIER.

**Methods:**

After 3 monthly loading doses, brolucizumab-treated eyes (*N* = 730) received injections every 12 weeks (q12w) or q8w if disease activity was detected. Aflibercept-treated eyes (*N* = 729) received fixed q8w dosing. Early persistent fluid was defined as the presence of subretinal fluid and/or intraretinal fluid up to Week 12.

**Results:**

A lower proportion of brolucizumab patients had early persistent retinal fluid compared with aflibercept (11.2% (*n* = 82) vs. 19.2% (*n* = 140)). In these patients, 34.1% of the brolucizumab-treated group achieved a ≥ 15 ETDRS letter gain in best corrected visual acuity (BCVA) from baseline at Week 96 compared with 20.7% of the aflibercept-treated group. Brolucizumab achieved numerically better BCVA outcomes (Week 96: brolucizumab, +6.4 letters; aflibercept, +3.7 letters) and significantly greater central subfield thickness reductions versus aflibercept from baseline through Week 96 (Week 96: −202 µm vs. −145 µm; *p* = 0.0206). Brolucizumab demonstrated an overall favourable benefit/risk profile in this patient cohort. In their unmasked, post-hoc review, the Safety Review Committee identified two cases of retinal vasculitis and no cases of retinal vascular occlusion in the brolucizumab arm; no cases of retinal vasculitis or retinal vascular occlusion were identified in the aflibercept arm.

**Conclusion:**

In this analysis, anatomical and visual outcomes were better with brolucizumab compared with aflibercept. Brolucizumab may therefore achieve greater disease control than aflibercept in nAMD patients with early persistent retinal fluid.

## Introduction

In neovascular age-related macular degeneration (nAMD), retinal fluid accumulation damages retinal structure and function and can potentially lead to vision loss and blindness, particularly if inadequately treated [1]. The current standard of care for the treatment of nAMD are anti-vascular endothelial growth factor (VEGF) therapeutics that inhibit the formation of new blood vessels, reduce retinal fluid accumulation and ultimately stabilise the retinal morphology [2, 3]. However, some patients have persistent retinal fluid despite monthly treatment with anti-VEGF injections, which may result in visual deterioration over time [4–6]. Therefore, these patients in particular need therapies that exhibit improved reductions in retinal fluid to optimise visual outcomes and reduce the overall treatment burden [7, 8].

Brolucizumab, a single-chain antibody fragment, allows for the delivery of more drug per dose and more VEGF-binding ability per volume compared with other currently available anti-VEGFs, and offers the potential for more effective tissue penetration and increased duration of action[9]. In the 2-year Phase III HAWK and HARRIER studies, brolucizumab 6 mg (administered in a q12w/q8w regimen) resulted in non-inferior best corrected visual acuity (BCVA) gains and superior anatomical outcomes versus aflibercept 2 mg (administered in a fixed q8w regimen), with over 50% of brolucizumab 6 mg patients maintained on a q12w treatment interval to Week 48 in patients with nAMD [10, 11]. The aim of the current post-hoc analysis is to compare the outcomes of brolucizumab 6 mg and aflibercept 2 mg treatment on BCVA and central subfield thickness (CST) in patients with early persistent retinal fluid from HAWK and HARRIER over the 96-week study period.

## Methods

### Study population and treatment

This is a post-hoc analysis of HAWK (NCT02307682) and HARRIER (NCT02434328), which were 96-week, randomised, double-masked, multicentre Phase III clinical studies [10, 11]. Protocols were approved by an Independent Ethics Committee/Institutional Review Board for each centre. Both studies were conducted in accordance with the principles of the Declaration of Helsinki, the International Conference on Harmonisation E6 Good Clinical Practice Consolidated Guideline, and other regulations as applicable, and were compliant with the US Health Insurance Portability and Accountability Act of 1996. All subjects provided written informed consent prior to screening or initiation of any study-related procedures.

Eligible patients were aged ≥50 years and had untreated active choroidal neovascularization (CNV) lesions secondary to age-related macular degeneration affecting the central subfield, intraretinal fluid (IRF) and/or subretinal fluid (SRF) affecting the central subfield as assessed on spectral-domain optical coherence tomography (SD-OCT), and BCVA between 23 and 78 Early Treatment Diabetic Retinopathy Study (ETDRS) letters. Full inclusion and exclusion criteria have been reported previously [11].

Eyes were randomised 1:1:1 to brolucizumab 3 mg, brolucizumab 6 mg, or aflibercept 2 mg (HAWK) or 1:1 to brolucizumab 6 mg or aflibercept 2 mg (HARRIER). As brolucizumab 6 mg is the approved dose for the treatment of nAMD, the brolucizumab 3 mg results will not be discussed further here. After injections at Weeks 0, 4 and 8 (loading phase), brolucizumab was injected q12w unless disease activity was identified at pre-specified disease activity assessment visits starting at Week 16, whereby treatment was adjusted to q8w for the remainder of the study; aflibercept was injected q8w, as per label at study initiation[11]. The patient subgroups for this analysis were defined post-randomisation based on baseline values and the patient response to treatment at Week 4, Week 8 and at the Week 12 monitoring visit. The ‘early persistent fluid’ subgroup was defined as those patients with IRF and/or SRF at baseline, Weeks 4, 8 and 12; the ‘early persistent SRF’ and ‘early persistent IRF’ subgroups were defined as the presence of SRF (with or without IRF) or IRF (with or without SRF), respectively, at all of the above time points.

### Clinical assessments and outcome measures

In HAWK and HARRIER, masked investigators conducted visual and anatomic assessments at baseline and every 4 weeks. BCVA was measured using ETDRS charts and SD-OCT imaging was used to measure CST, and the presence of IRF/SRF in the central subfield (6 × 6 mm macula centred). In both studies, SD-OCT images were evaluated by central reading centres (Duke Reading Centre, Durham, NC, USA for HAWK; Vienna Reading Centre, Vienna, Austria for HARRIER).

Outcomes through the 96-week follow-up period in this post-hoc analysis are presented here as follows: the proportion of eyes that lost or gained ≥15 ETDRS letters; mean change in BCVA from baseline to Week 96 in the whole early persistent fluid subgroup and in those patients with early persistent SRF (with or without IRF) or early persistent IRF (with or without SRF); and mean change in CST from baseline to Week 96. The proportions of patients with retinal fluid at Weeks 16, 48 and 96 are also presented, in addition to key safety outcomes in this subgroup.

### Statistical analyses

Differences in BCVA and CST outcomes between brolucizumab 6 mg and aflibercept 2 mg patients from the pooled HAWK and HARRIER data were analysed using the analysis of variance (ANOVA) model with baseline BCVA/CST and age categories as covariates. Missing values were imputed using the last observed value carried forward (LOCF) method. All *p*-values are 2-sided and are not adjusted for multiplicity. We consider *p*-values below 0.05 to be statistically significant.

### Brolucizumab safety review committee

In early 2020, following post-marketing reports of vasculitis, including retinal occlusive vasculitis, associated with intraocular inflammation (IOI) with brolucizumab, Novartis convened an external Safety Review Committee (SRC) to provide an independent review of these cases and a comparison with events seen in the HAWK and HARRIER trials. The SRC performed an unmasked post-hoc review of all cases of investigator-reported IOI (including the case of perivascular sheathing), retinal vascular occlusions and endophthalmitis, including those occurring in patients with early persistent retinal fluid [12].

## Results

### Patient population

**Fig. 1:**
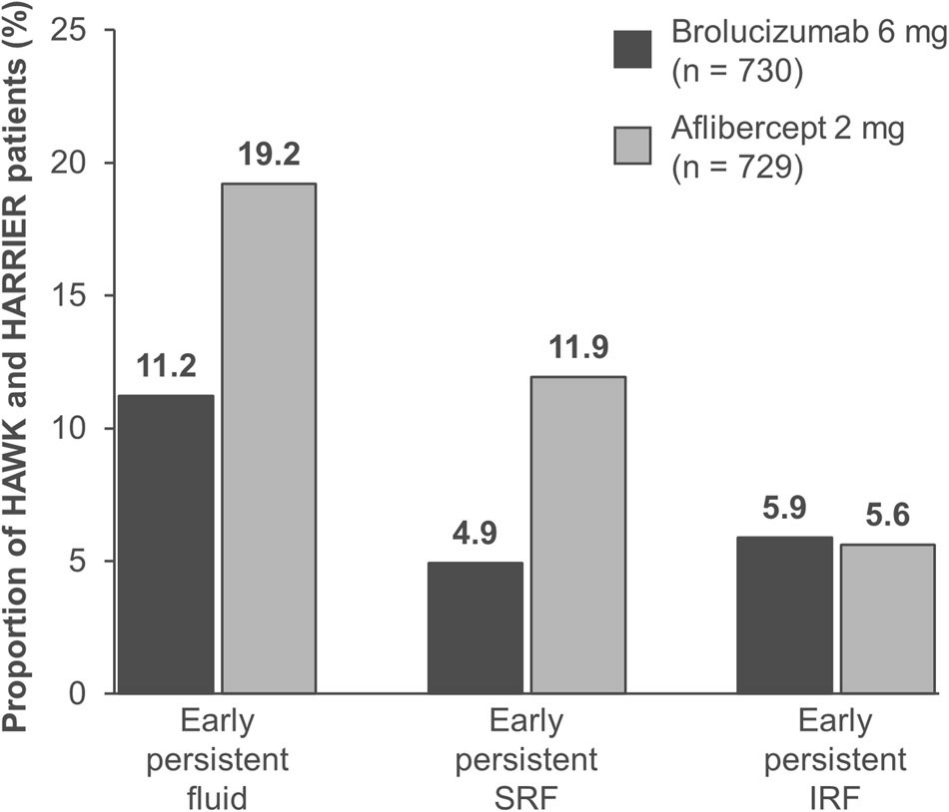
Proportions of HAWK and HARRIER patients with early persistent fluid (IRF and/or SRF), early persistent SRF (with or without IRF) and early persistent IRF (with or without SRF) at baseline and at Weeks 4, 8 and 12. Note that the ‘early persistent SRF’ group comprises subjects with the presence of SRF at all study visits through Week 12 and likewise for the ‘early persistent IRF’ group whereas the ‘early persistent fluid’ group includes subjects with either fluid at these visits. IRF intraretinal fluid, SRF subretinal fluid.

In the pooled treatment arms in HAWK & HARRIER, the proportion of patients with early persistent fluid (IRF and/or SRF) was lower in the brolucizumab 6 mg group (11.2% [82/730 patients]) than in the aflibercept 2 mg group (19.2% [140/729 patients]). Similarly, the proportion of patients with persistent SRF (with or without IRF) was lower in the brolucizumab 6 mg group (4.9% [36/730 patients]) than in the aflibercept 2 mg group (11.9% [87/729 patients]) whereas the proportions of patients with persistent IRF (with or without SRF) were comparable in both groups (brolucizumab 6 mg, 5.9% [43/730 patients]; aflibercept 2 mg, 5.6% [41/729 patients]) (Fig. 1).

Demographic and clinical characteristics at baseline were well-balanced between groups with regards to age and gender (Supplementary Table 1). There were slight differences in mean BCVA and CST; however, these imbalances were accounted for in the ANOVA model. Study discontinuation rates were higher in the aflibercept-treated group (*n* = 27; 19.3%) than in the brolucizumab-treated group (*n* = 7; 8.5%). The primary reasons for discontinuation in the aflibercept group were ‘progressive disease’ and ‘withdrawal by subject’ (6 patients each) whereas ‘lost to follow up’ was the most common reason in the brolucizumab group (*n* = 2) (Supplementary Table 2).

### Visual outcomes

**Fig. 2:**
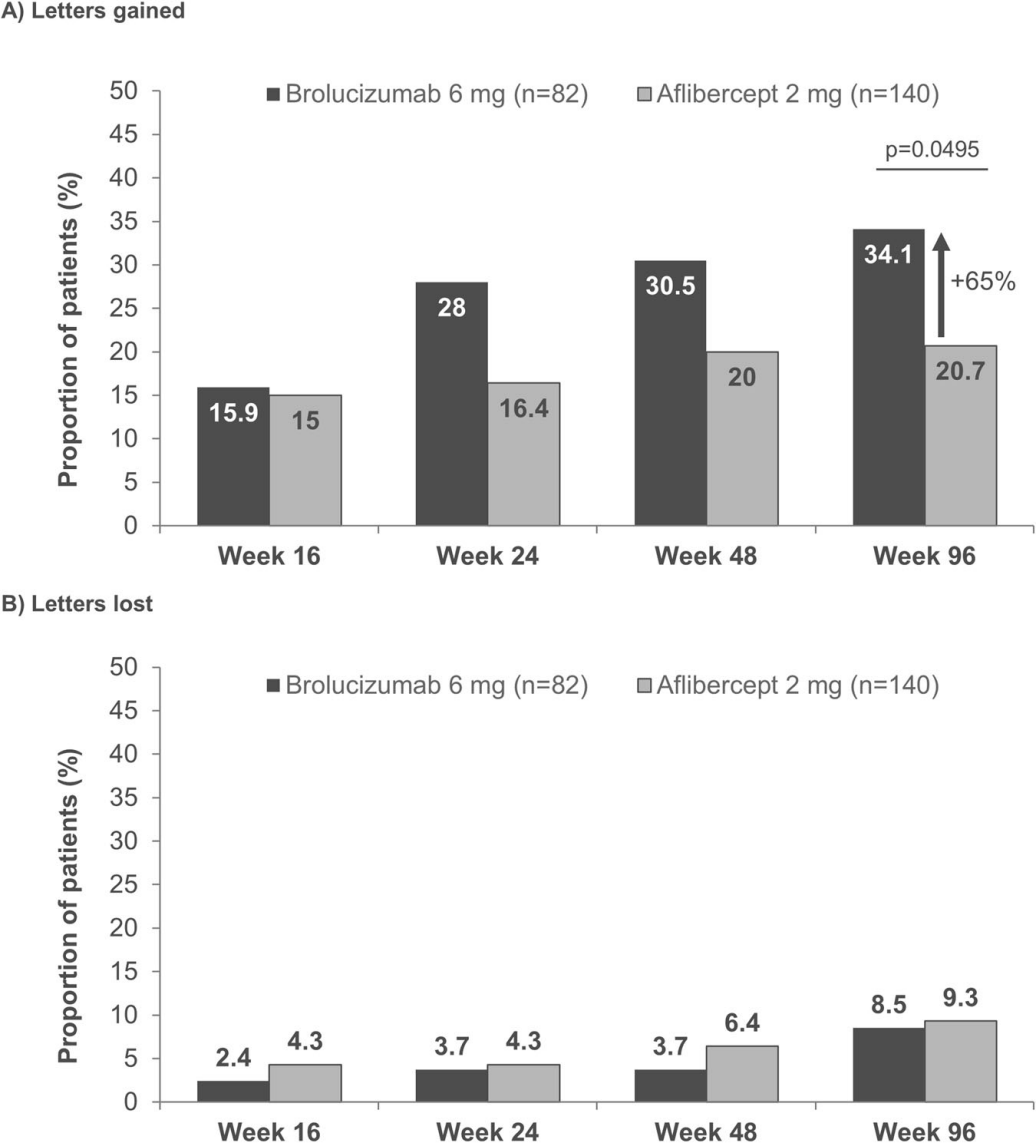
Proportion of early persistent fluid patient eyes gaining (**A**) or losing (**B**) ≥15 ETDRS letters from baseline at Weeks 16, 24, 48 and 96. Analysed using ANOVA model with baseline BCVA categories (< = 55, 56–70, > = 71 letters), age categories (<75, ≥75 years) and treatment as fixed effect factors. The percent relative difference in proportion of patients who gained ≥15 ETDRS letters at Week 96 is calculated with aflibercept as a reference. ANOVA analysis of variance, ETDRS Early Treatment Diabetic Retinopathy Study.

The proportion of patients with early persistent fluid who achieved a ≥ 15 ETDRS letter gain in BCVA from baseline was higher with brolucizumab 6 mg compared with aflibercept 2 mg, respectively, at Week 16 (15.9% vs. 15.0%), Week 24 (28.0% vs 16.4%), Week 48 (30.5% vs. 20.0%), and Week 96 (34.1% vs. 20.7%, *p* = 0.0495). The percent relative difference in the proportion of patients who gained ≥15 ETDRS letters at Week 96 was +65% for brolucizumab compared with aflibercept (Fig. 2A). No notable differences were observed in the proportion of patients losing ≥15 letters through to Week 96 (Fig. 2B).

**Fig. 3:**
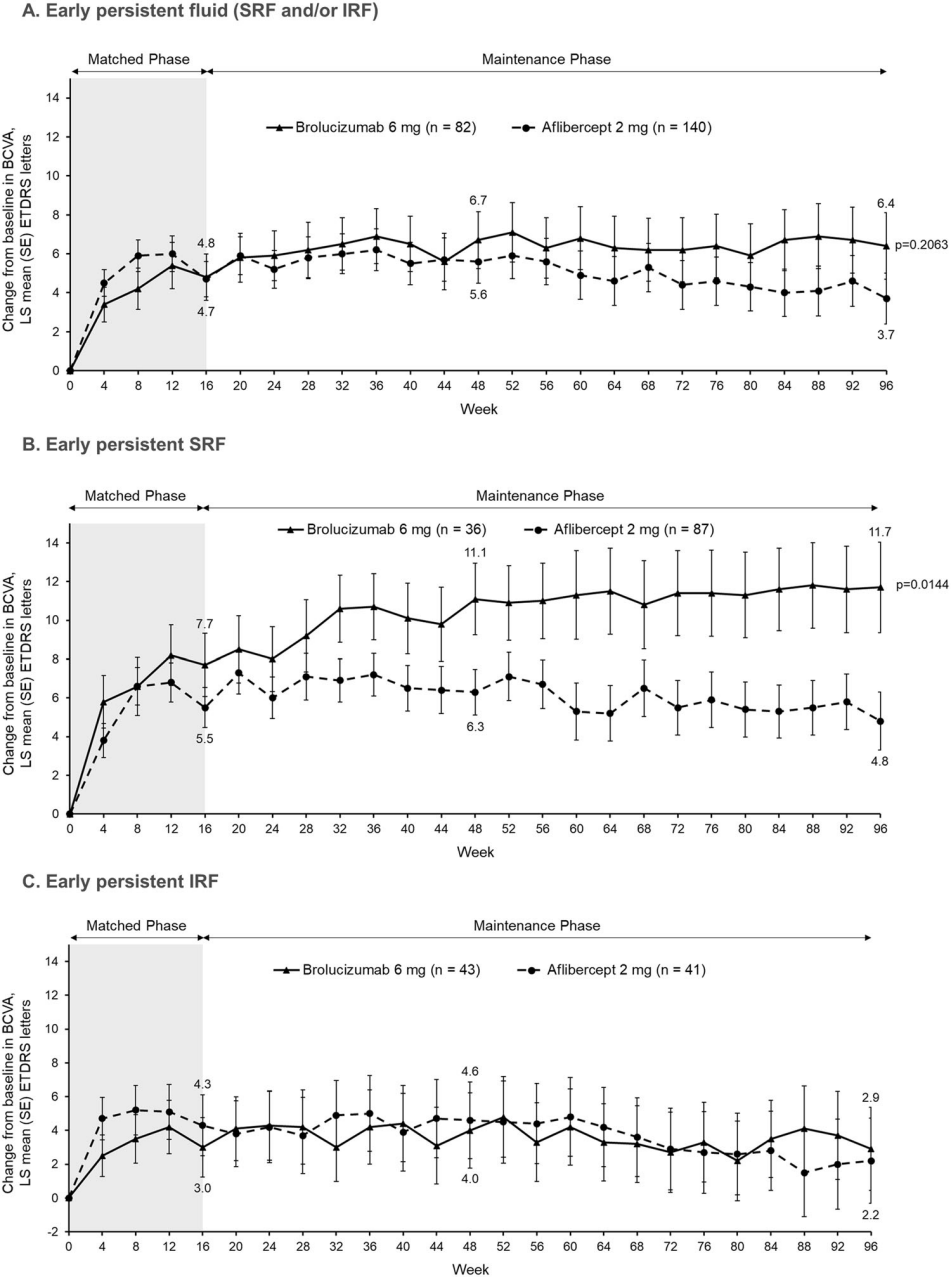
Change in BCVA from baseline through to Week 96 in eyes with persistent retinal fluid. **A** BCVA changes in eyes with persistent IRF and/or SRF (aflibercept-treated eyes: *n* = 140; brolucizumab-treated eyes: *n* = 82); **B** BCVA changes in eyes with persistent SRF (with or without IRF; aflibercept-treated eyes: *n* = 87; brolucizumab-treated eyes: *n* = 36); **C** BCVA changes in eyes with persistent IRF (with or without SRF; aflibercept-treated eyes: *n* = 41; brolucizumab-treated eyes: *n* = 43). Analysed using ANOVA model with baseline BCVA categories (< = 55, 56–70, > = 71 letters), age categories (<75, ≥75 years) and treatment as fixed effect factors. ANOVA analysis of variance, BCVA best corrected visual acuity, ETDRS Early Treatment Diabetic Retinopathy Study, IRF intraretinal fluid, LS least squares, SE standard error; SRF subretinal fluid.

Overall, patients with early persistent fluid (defined as IRF and/or SRF from baseline through to Week 12) had numerically better BCVA outcomes with brolucizumab compared with aflibercept, as the least square (LS) mean (standard error) BCVA gains at Weeks 48 and 96 were +6.7 (1.5) and +6.4 (1.7) letters respectively with brolucizumab compared with +5.6 (1.1) and +3.7 (1.3) letters respectively with aflibercept (Fig. 3A). In eyes with persistent SRF (with or without IRF), robust BCVA improvements were observed already by Week 32 in eyes treated with brolucizumab compared with aflibercept-treated eyes and at Week 96, the difference between the two treatment arms was significant (brolucizumab + 11.7 (2.3) letters vs aflibercept +4.8 (1.5) letters with a difference of 6.9 letters (95% confidence interval (CI): [1.4, 12.5]), *p* = 0.0144) (Fig. 3B). BCVA outcomes were comparable in both treatment arms for eyes with persistent IRF (with or without SRF) (Fig. 3C).

### Anatomic outcomes

**Fig. 4:**
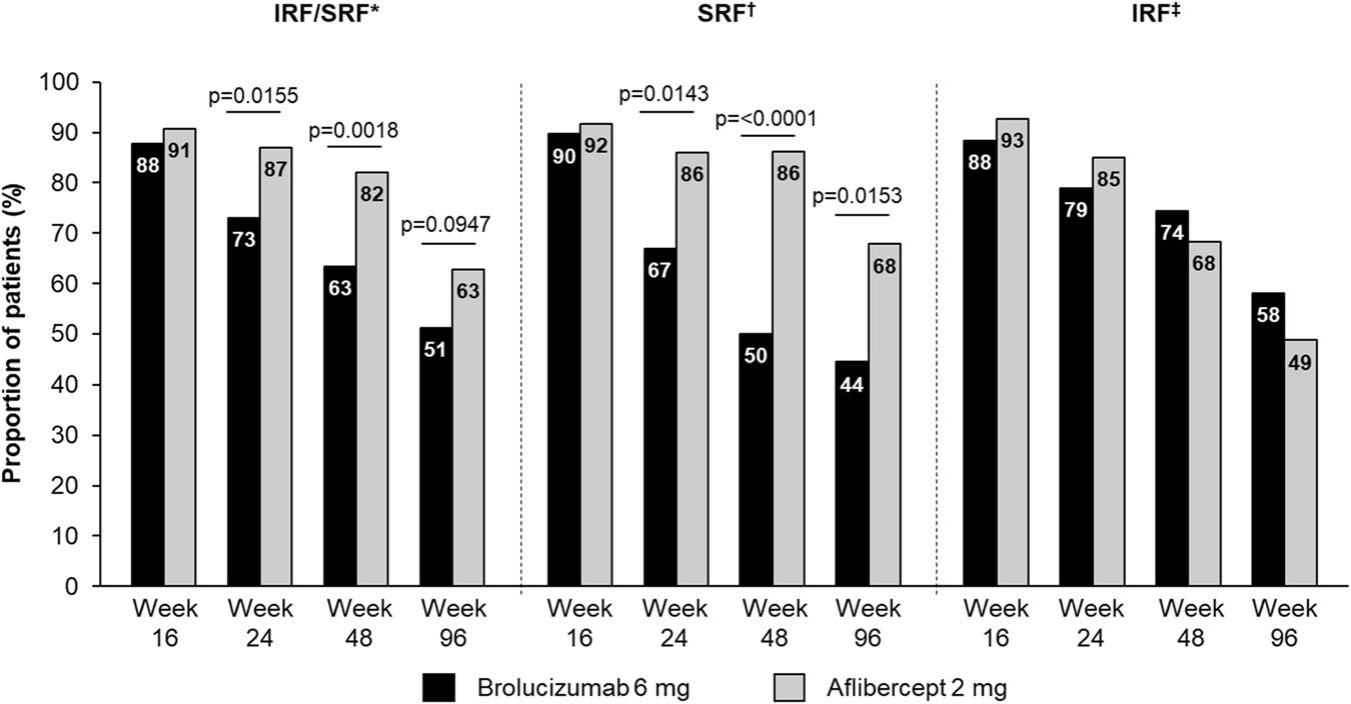
Proportion of patients IRF and/or SRF, SRF or IRF at Weeks 16, 48 and 96 among patients with early persistent retinal fluid. IRF intraretinal fluid, SRF subretinal fluid. *Proportion of 140 eyes treated with aflibercept and 82 eyes treated with brolucizumab, respectively. ^†^Proportion of 87 eyes treated with aflibercept and 36 eyes treated with brolucizumab, respectively. ^‡^Proportion of 41 eyes treated with aflibercept and 43 eyes treated with brolucizumab, respectively.

Among the patients with early persistent fluid, the proportions of patients with retinal fluid (IRF, SRF, IRF and/or SRF) decreased with time in both treatment groups (Fig. 4). The proportions of patients with SRF decreased significantly more in the brolucizumab-treated group compared with the aflibercept-treated group (Week 24: 67% vs 86%, *p* = 0.0143; Week 48: 50% vs 86%, *p* = <0.0001; Week 96: 44% vs 68%, *p* = 0.0153). By contrast, the proportions of patients with presence of IRF at Weeks, 16, 24, 48, and 96 were comparable among the two treatments.

**Fig. 5:**
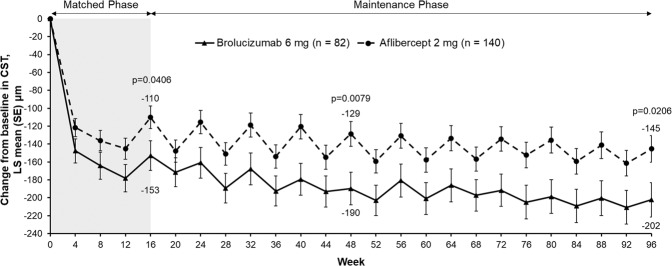
Change in CST from baseline through to Week 96 in eyes with early persistent fluid (IRF and/or SRF). Analysed using ANOVA model with baseline CST-total categories (<400, > = 400 µm), age categories (<75, > = 75 years) and treatment as fixed effect factors. ANOVA analysis of variance, CST central subfield thickness, LS least squares, SE standard error.

Brolucizumab achieved consistently greater CST reductions in the subgroup with early persistent fluid (IRF and/or SRF) from baseline versus aflibercept beginning after the first injection at Week 4 (LS mean −147 [13] µm vs. −122 [10] µm), and this reduction was maintained throughout the study. The LS mean change in CST (µm) from baseline was −153 (16) vs. −110 (13) at Week 16 (treatment difference −43, 95% CI [−84, −2], *p* = 0.0406); −190 (18) vs. −129 (14) at Week 48 (treatment difference −61, 95% CI [−106, −16], *p* = 0.0079), and −202 (19) vs. −145 (15) at Week 96 (treatment difference −57, 95% CI [−105, −9], *p* = 0.0206) in the brolucizumab and aflibercept groups, respectively (Fig. 5).

### Safety outcomes

Ocular adverse events occurring in ≥3% of patients with early persistent fluid and all serious ocular adverse events are presented in Supplementary Table 3. One serious adverse event of endophthalmitis occurred in each treatment group: in the aflibercept arm, the case was culture positive and at the end of study, the patient lost 51 letters compared with baseline; the brolucizumab case was culture negative and the patient lost 11 letters by end of study compared with baseline. With regards to investigator-reported IOI, there was one case of uveitis in the brolucizumab 6 mg arm, the severity of which was classed as moderate and the patient gained 16 letters by the end of study.

Following their review of the post-marketing cases and the Phase III studies, the SRC identified a spectrum of inflammatory signs ranging from IOI to retinal vasculitis to retinal vascular occlusion that sometimes resulted in visual acuity loss [12]. In both the uveitis and endophthalmitis case in the brolucizumab arm, the SRC identified signs of retinal vasculitis but not retinal vascular occlusion.

## Discussion

This post-hoc analysis of the HAWK & HARRIER studies has shown that a lower proportion of patients treated with brolucizumab had early persistent fluid, defined as IRF and/or SRF present at baseline and at Weeks 4, 8 and 12, compared with those treated with aflibercept. In this cohort with early persistent fluid, the proportion of patients who gained ≥15 letters was significantly higher in the brolucizumab-treated group at Week 96, and numerically better BCVA outcomes were observed with brolucizumab compared with aflibercept, particularly in patients with persistent SRF (with or without IRF). Furthermore, greater CST reductions were also seen early and consistently with brolucizumab compared with aflibercept.

Although the patients with early persistent fluid were likely to have had a high treatment need, the brolucizumab-treated patients in this cohort would have been a heterogeneous group with regards to treatment interval. The first disease activity assessment visit to identify which patients were suitable for q12w dosing took place at Week 16. Protocol guidance was provided but ultimately, treatment decisions were made by the masked investigator based on their own clinical judgement. Some patients in this cohort may therefore have been judged to be eligible for q12w dosing depending on functional and anatomical parameters other than fluid.

The results of this study are validated by the post-hoc analysis of the VIEW 1 and 2 trials, in which the effects of aflibercept and ranibizumab on visual acuity outcomes in nAMD eyes with early persistent retinal fluid after three initial monthly injections were evaluated. This study found that 20.3% and 29.4% of eyes treated with aflibercept q8w or ranibizumab q4w, respectively, had persistent fluid present at baseline and at follow-ups at Weeks 4, 8 and 12 [4]. In the present analysis, 11.2% of patients treated with brolucizumab had persistent retinal fluid (i.e., fluid present at baseline and at Weeks 4, 8 and 12) compared with 19.2% of patients treated with aflibercept. The proportion of aflibercept-treated eyes with persistent fluid is remarkably similar between the VIEW 1 and VIEW 2 and the HAWK and HARRIER post-hoc analyses cohorts (20.3% vs. 19.2%).

The proportion of patients who gained ≥15 letters was significantly higher in the brolucizumab-treated group at Week 96 and although not statistically significant, overall visual outcomes in patients with early persistent fluid were numerically better with brolucizumab at Week 96 (LS mean change from baseline of +6.4 letters in the brolucizumab arm compared with +3.7 letters in the aflibercept arm). Nevertheless, the change in BCVA graphs between brolucizumab and aflibercept appear to separate through to Week 48 and that separation increases to Week 96. Eyes with persistent fluid typically require ongoing anti-VEGF treatment beyond Week 96. Therefore, there may be further long-term benefit to visual acuity with more prolonged brolucizumab treatment, although more data are needed to confirm this hypothesis.

It is not surprising that the mean BCVA gains at Week 96 are similar in the two treatment arms in patients with persistent IRF, as the data presented here also show that brolucizumab is comparable to aflibercept with respect to resolution of IRF. By contrast, the significantly better visual outcomes in brolucizumab-treated patients with persistent SRF correspond to the observed greater resolution of SRF in these patients. These data are also consistent with the observation in the Phase II OSPREY study that brolucizumab is better at resolving SRF than aflibercept [13]. At 26 kDa, the small size of brolucizumab and the ability to administer more drug per dose may facilitate rapid and more effective penetration of the different retinal layers [9]. This could enable brolucizumab to dry the retina more at the source of nAMD, thus preventing the migration of fluid into the more anterior spaces where it has potential to have a detrimental effect on visual function [14]. The potential association between SRF resolution and higher VA gains observed here is in agreement with a real-world study in which there was a positive correlation between the number of clinic visits during the anti-VEGF maintenance phase with an absence of SRF and gain in VA at Month 12 [15]. Furthermore, a post-hoc analysis of the FLUID study showed that the presence of SRF in the central 1−6 mm macular area was negatively associated with BCVA [16].

Data from the primary analysis of the HAWK and HARRIER studies suggested that brolucizumab is more effective at resolving retinal fluid than aflibercept in patients with nAMD [10], and this is supported by the fact that a lower proportion of patients in the brolucizumab group met the inclusion criteria of this post-hoc analysis with retinal fluid at all visits to Week 12. Brolucizumab-treated eyes with early persistent fluid may therefore have more severe disease and be more difficult to treat than those selected with aflibercept. Nevertheless, brolucizumab patients in this subgroup had better BCVA gains and better fluid control and hence better disease control overall than aflibercept-treated patients. In accordance with these observations, more patients with early persistent fluid in the aflibercept arm (19.3%) discontinued from HAWK and HARRIER because of a lack of efficacy or progressive disease than those treated with brolucizumab (8.5%).

With regards to the safety outcomes with brolucizumab and aflibercept, no ocular adverse events or serious ocular adverse events in the persistent retinal fluid cohorts were found to affect the efficacy analysis of this post-hoc study. Two cases of vasculitis were identified in the early persistent fluid patient cohort by the SRC, neither of which were associated with severe vision loss.

The main strength of this post-hoc analysis is that it is based on data from two large, double-blinded Phase III trials. Limitations include that the analysed patient cohorts were based on patients’ response to treatment and were not randomised specifically for this post-hoc analysis, and that the number of patients in each treatment group was relatively small.

The presence of early persistent retinal fluid in patients with nAMD despite 3 monthly anti-VEGF injections is indicative of particularly aggressive disease. This analysis suggests that in such patients, brolucizumab may achieve greater disease control than aflibercept, which ultimately may translate into lower long-term treatment burden and more optimal visual outcomes.

## Summary

### What was known before

- Some patients with nAMD have persistent retinal fluid despite monthly treatment with anti-VEGF injections, which may result in visual deterioration over time.
- Therefore, these patients in particular need therapies that exhibit improved reductions in retinal fluid to optimise visual outcomes and reduce the overall treatment burden.

### What this study adds

- This post-hoc analysis shows that in HAWK and HARRIER, a lower proportion of patients treated with brolucizumab 6 mg had early persistent retinal fluid compared with those treated with aflibercept.
- In this subgroup with early persistent fluid, brolucizumab patients had better BCVA gains and better fluid control than aflibercept-treated patients.
- Brolucizumab may therefore achieve greater disease control in nAMD patients with early persistent fluid than aflibercept.

## Supplementary information

Supplementary Table 1

Supplementary Table 2

Supplementary Table 3

## Data Availability

All data generated or analysed during this study are included in this published article (and its Supplementary Information Files).
