# Correction: Efficacy and safety of brolucizumab versus aflibercept in eyes with early persistent retinal fluid: 96-week outcomes from the HAWK and HARRIER studies

**DOI:** 10.1038/s41433-022-02130-2

**Published:** 2022-06-08

**Authors:** David R. Lally, Anat Loewenstein, Jennifer J. Arnold, Yit C. Yang, Kinfemichael Gedif, Catherine Best, Hersh Patel, Ramin Tadayoni, Jeffrey S. Heier

**Affiliations:** 1 New England Retina Consultants, Springfield, MA USA; 2 Department of Surgery, University of Massachusetts Medical School-Baystate, Springfield, MA USA; 3 Tel Aviv Sourasky Medical Center, Sackler Faculty of Medicine, Tel Aviv University, Tel Aviv, Israel; 4 Marsden Eye Specialists, Parramatta, NSW Australia; 5 Royal Wolverhampton Hospitals NHS Trust, Wolverhampton, UK; 6 Novartis Pharmaceuticals Corporation, Fort Worth, TX USA; 7 Novartis Pharma AG, Basel, Switzerland; 8 Novartis Pharmaceuticals Corporation, East Hannover, NJ USA; 9 Department of Ophthalmology, Université de Paris, AP-HP, Lariboisière, Saint Louis and Rothschild Foundation Hospitals, Paris, France; 10 Ophthalmic Consultants of Boston, Boston, MA USA; 11 Present Address: IVERIC bio, Inc., New York, NY USA

Correction to: *Eye* 10.1038/s41433-022-02092-5, published online 21 May 2022

The article “Efficacy and safety of brolucizumab versus aflibercept in eyes with early persistent retinal fluid: 96-week outcomes from the HAWK and HARRIER studies”, written by David R. Lally, Anat Loewenstein, Jennifer J. Arnold, Yit C. Yang, Kinfemichael Gedif, Catherine Best, Hersh Patel, Ramin Tadayoni, Jeffrey S. Heier, was originally published electronically on the publisher’s internet portal on 21 May 2022 without open access. With the author(s)’ decision to opt for Open Choice the copyright of the article changed on 30 May 2022 to © The Author(s) 2022 and the article is forthwith distributed under a Creative Commons Attribution 4.0 International License, which permits use, sharing, adaptation, distribution and reproduction in any medium or format, as long as you give appropriate credit to the original author(s) and the source, provide a link to the Creative Commons licence, and indicate if changes were made. The images or other third-party material in this article are included in the article’s Creative Commons licence, unless indicated otherwise in a credit line to the material. If material is not included in the article’s Creative Commons licence and your intended use is not permitted by statutory regulation or exceeds the permitted use, you will need to obtain permission directly from the copyright holder. To view a copy of this licence, visit http://creativecommons.org/licenses/by/4.0/.

