# Supplementary material for: Efficacy and safety of brolucizumab versus aflibercept in eyes with early persistent retinal fluid: 96-week outcomes from the HAWK and HARRIER studies
Source: Eye (Lond). 2022 May 21;37(6):1242–8. doi: 10.1038/s41433-022-02092-5 (PMC10102125; doi:10.1038/s41433-022-02092-5)
Supplement: Supplementary file 1 — Supplementary Table 1 [file 41433_2022_2092_MOESM1_ESM.pdf]

Supplementary Table 1. Demographic and clinical characteristics at baseline of patients with early persistent retinal fluid

Characteristics	Brolucizumab 6 mg N=82 patients	Aflibercept 2 mg N=140 patients
**Age at index (patient, years)**		
Mean (SD)	75.6 (9.7)	74.4 (7.7)
**Gender (patient; n, %)**		
Male	44 (53.7)	79 (56.4)
Female	38 (46.3)	61 (43.6)
**nAMD laterality (patient; n, %)**		
Unilateral	67 (81.7%)	114 (81.4%)
Bilateral	15 (18.3%)	26 (18.6%)
**BCVA (ETDRS letters)**		
Mean (SD)	53.1 (16.6)	58.8 (14.3)
≤55 letters (n, %)	40 (48.8)	50 (35.7)
56–70 letters (n, %)	29 (35.4)	60 (42.9)
≥71 letters (n, %)	13 (15.9)	30 (21.4)
**CST (µm)**		
Mean (SD)	535.0 (184.8)	479.4 (151.5)
<400 µm (n, %)	20 (24.4)	44 (31.4)
≥400 µm (n, %)	62 (75.6)	96 (68.6)
**CNV type**		
Predominantly classic	42 (51.2)	56 (40.0)
Minimally classic	7 (8.5)	8 (5.7)
Occult	33 (40.2)	76 (54.3)
**Area of lesion associated with CNV, mm^2 (SD)**	4.5 (4.6)	4.1 (4.0)
**CNV location**		
Subfoveal	57 (69.5)	97 (69.3)
Juxtafoveal	17 (20.7)	26 (18.6)
Extrafoveal	8 (9.8)	17 (12.1)

**IRF (n, %)**		
Present	52 (63.4)	65 (46.4)
Absent	30 (36.6)	75 (53.6)
**SRF (n, %)**		
Present	58 (70.7)	113 (80.7)
Absent	24 (29.3)	27 (19.3)
**Sub-RPE fluid (n, %)**		
Present	33 (40.2)	58 (41.4)
Absent	49 (59.8)	82 (58.6)
**Intraretinal hemorrhage (n, %)**		
Present	15 (18.3)	11 (7.9)
Absent	67 (81.7)	129 (92.1)
**Subretinal hemorrhage (n, %)**		
Present	10 (12.2)	14 (10.0)
Absent	72 (87.8)	126 (90.0)

BCVA, best corrected visual acuity; CNV, choroidal neovascularization; CST, central subfield thickness; ETDRS, Early Treatment Diabetic Retinopathy Study; IRF, Intraretinal fluid; nAMD, neovascular age-related macular degeneration; RPE, retinal pigment epithelium; SD, standard deviation; SRF, subretinal fluid.
