# Supplementary material for: Efficacy and safety of brolucizumab versus aflibercept in eyes with early persistent retinal fluid: 96-week outcomes from the HAWK and HARRIER studies
Source: Eye (Lond). 2022 May 21;37(6):1242–8. doi: 10.1038/s41433-022-02092-5 (PMC10102125; doi:10.1038/s41433-022-02092-5)
Supplement: Supplementary file 2 — Supplementary Table 2 [file 41433_2022_2092_MOESM2_ESM.pdf]

Supplementary Table 2. Patient disposition to Week 96 in patients with early persistent retinal fluid

Reason for discontinuation	Brolucizumab 6 mg N=82; n (%)	Aflibercept 2 mg N=140; n (%)
Adverse event	1 (1.2)	4 (2.9)
Death	1 (1.2)	3 (2.1)
Lack of efficacy	0 (0.0)	4 (2.9)
Lost to follow-up	2 (2.4)	3 (2.1)
Physician decision	0 (0.0)	1 (0.7)
Progressive disease	0 (0.0)	6 (4.3)
Withdrawal by subject	1 (1.2)	6 (4.3)
Other	2 (2.4)	0 (0.0)
**Total**	**7 (8.5)**	**27 (19.3)**
