# Supplementary material for: Efficacy and safety of brolucizumab versus aflibercept in eyes with early persistent retinal fluid: 96-week outcomes from the HAWK and HARRIER studies
Source: Eye (Lond). 2022 May 21;37(6):1242–8. doi: 10.1038/s41433-022-02092-5 (PMC10102125; doi:10.1038/s41433-022-02092-5)
Supplement: Supplementary file 3 — Supplementary Table 3 [file 41433_2022_2092_MOESM3_ESM.pdf]

Supplementary Table 3. Ocular adverse events and serious ocular adverse events in eyes with persistent retinal fluid

Primary system organ class / Preferred term	Brolucizumab 6 mg N=82; n (%)	Aflibercept 2 mg N=140; n (%)
**Ocular adverse events reported in ≥3% of study participants in either treatment group**		
Number of subjects with at least one event	40 (48.8)	75 (53.6)
Eye disorders	39 (47.6)	71 (50.7)
Visual acuity reduced	7 (8.5)	14 (10.0)
Conjunctival hemorrhage	5 (6.1)	13 (9.3)
Blepharitis	5 (6.1)	4 (2.9)
Vitreous floaters	5 (6.1)	2 (1.4)
Retinal hemorrhage	4 (4.9)	10 (7.1)
Eye pain	4 (4.9)	7 (5.0)
Cataract	3 (3.7)	5 (3.6)
Chalazion	3 (3.7)	1 (0.7)
Retinal pigment epithelial tear	3 (3.7)	1 (0.7)
Dry eye	2 (2.4)	9 (6.4)
Vision blurred	1 (1.2)	5 (3.6)
Vitreous detachment	1 (1.2)	5 (3.6)
Injury, poisoning and procedural complications	4 (4.9)	6 (4.3)
Corneal abrasion	3 (3.7)	4 (2.9)
Investigations	3 (3.7)	6 (4.3)
Intraocular pressure increased	3 (3.7)	5 (3.6)
**Serious ocular adverse events**		
Number of subjects with at least one serious ocular adverse event	3 (3.7)	5 (3.6)
Eye disorders	2 (2.4)	4 (2.9)
Macular hole	1 (1.2)	1 (0.7)
Retinal tear	1 (1.2)	0 (0.0)

Visual acuity reduced	0 (0.0)	2 (1.4)
Retinal detachment	0 (0.0)	1 (0.7)
Infections and infestations	1 (1.2)	1 (0.7)
Endophthalmitis	1 (1.2)	1 (0.7)
